# Structured Data Capture for Oncology

**DOI:** 10.1200/CCI.20.00103

**Published:** 2021-02-16

**Authors:** Alexander K. Goel, Walter Scott Campbell, Richard Moldwin

**Affiliations:** ^1^ Cancer Protocols and Data Standards, College of American Pathologists, Northfield, IL; ^2^ Department of Pathology/Microbiology, University of Nebraska Medical Center, Omaha, NE

## Abstract

Lack of interoperability is one of the greatest challenges facing healthcare informatics. Recent interoperability efforts have focused primarily on data transmission and generally ignore data capture standardization. Structured Data Capture (SDC) is an open-source technical framework that enables the capture and exchange of standardized and structured data in interoperable data entry forms (DEFs) at the point of care. Some of SDC’s primary use cases concern complex oncology data such as anatomic pathology, biomarkers, and clinical oncology data collection and reporting. Its interoperability goals are the preservation of semantic, contextual, and structural integrity of the captured data throughout the data’s lifespan. SDC documents are written in eXtensible Markup Language (XML) and are therefore computer readable, yet technology agnostic—SDC can be implemented by any EHR vendor or registry. Any SDC-capable system can render an SDC XML file into a DEF, receive and parse an SDC transmission, and regenerate the original SDC form as a DEF or synoptic report with the response data intact. SDC is therefore able to facilitate interoperable data capture and exchange for patient care, clinical trials, cancer surveillance and public health needs, clinical research, and computable care guidelines. The usability of SDC-captured oncology data is enhanced when the SDC data elements are mapped to standard terminologies. For example, an SDC map to Systematized Nomenclature of Medicine Clinical Terms (SNOMED CT) enables aggregation of SDC data with other related data sets and permits advanced queries and groupings on the basis of SNOMED CT concept attributes and description logic. SDC supports terminology maps using separate map files or as terminology codes embedded in an SDC document.

## INTRODUCTION

Interoperability, in the context of complex oncology data sets, is the ability to share and reuse data across multiple nodes without semantic, contextual, or structural loss.^1^ Reuse of data refers primarily to secondary usage in an external data ecosystem for purposes such as patient care, cancer surveillance, research, and clinical trials. Interoperability is greatly enhanced by standardizing the structure of contextually related data fields, before capturing in an electronic health record (EHR) system.

**CONTEXT**

**Key Objective**
Review the current state of the Structured Data Capture (SDC) initiative in the oncology data ecosystem.
**Knowledge Generated**
SDC is a computer-readable information model that defines the information content of data-entry forms, supports multiple approaches for SDC data exchange, and enables secondary use of standardized data.
**Relevance**
SDC templates allow clinical information to be standardized for data capture before entry into computer systems. SDC is especially valuable for the collection and exchange of rapidly versioned data elements such as those found in pathology data sets, cancer staging, and clinical trials.

For patient care, preservation of structure and context is critical, from the data entry form (DEF) through all downstream clinical reports. Centralized standardization of data entry fields during the data collection design process, with a focus on downstream interoperability and data reuse, has several benefits.^2^ The design of data fields and DEF structure by centralized expert teams can make data entry more consistent and efficient, aiding in the data entry process. Standardization of data entry with consistent evidence-based data fields helps to ensure complete collection of clinically critical data in a familiar format and enables the generation of consistent, standardized, and structured reports, regardless of EHR vendor, institution, or variations in the cosmetics of DEF and report formats.^3-5^

Unfortunately, this type of precapture standardization is rarely addressed by EHR vendors. Attempts to standardize and/or aggregate data fields across EHRs after the data are collected often require a significant effort in data aggregation and cleaning and often yield suboptimal results.^6,7^ The lack of precapture semantic, contextual, and structural standardization is thus a significant barrier to the complex data analyses required in oncology investigations and is a barrier to sharing data with patients, their care teams, and other EHR systems.^8^

Structured Data Capture (SDC) is an open-source technical framework published by the Quality Research and Public Health committee of the standards organization Integrating the Healthcare Enterprise (IHE). SDC was designed to solve the problem of precapture data standardization in an interoperable manner. SDC can be viewed as a model that specifies the structure of related data elements (DEs) and preserves their semantic and contextual integrity. Furthermore, SDC specifies the information content of interoperable DEFs so that the DEF user can capture, store, and exchange complex, context-rich data in standardized DEs.^9^ An SDC template specifies the content of a DEF that can be rendered by any EHR vendor in a technology-agnostic manner, while maintaining an exact representation of the data definitions, allowing the captured data to be exchanged in an interoperable manner. SDC-based DEFs are particularly useful for designing and exchanging complex oncology data sets, such as those needed for anatomic pathology, biomarkers, and clinical oncology reporting.

Since 2019, SDC has been the delivery format for the electronic Cancer Checklists (eCCs) from the College of American Pathologists (CAPs). These checklists are used by 35%-40% of North American pathologists.^10,11^ Much of the data captured by these forms are submitted to North American cancer registries for public health surveillance.^12,13^ Other clinical specialties (eg, radiology and surgery) are exploring the use of SDC for standardizing data entry, delivering standardized clinical reports, and facilitating downstream data usages. The eCC program is described in another paper in this issue.^10^

## SDC HISTORY

The SDC project was initiated in early 2013 by the Office of the National Coordinator for Health Information Technology (ONC) through its Standards and Interoperability Framework initiative.^14^ IHE was selected as the organization to host the specification. The IHE profile for SDC was first published in October 2016, is maintained by the IHE SDC Working Group, and is regularly tested at IHE Connectathons.^15,16^

The ONC also sponsored an attempt to harmonize FHIR Questionnaire with IHE SDC,^17^ to produce a hybrid, functionally equivalent FHIR SDC model. However, complete harmonization was not achieved, and the two approaches diverged because of differences in objectives and design principles. In 2017, both IHE SDC and FHIR SDC became community-led initiatives. This paper addresses only IHE SDC.

## SDC ARCHITECTURE

SDC is an information model that describes how various types of generic clinical information should be represented for technology-agnostic data capture. The primary information type addressed by SDC is the DE,^18^ which includes question-answer sets and fill-in questions, although SDC can also handle standard media types such as images in questions and responses. Each question and answer has a unique identifier (ID), which remains constant unless the contextual semantics of the question or answer changes. To help represent context and control the display of form parts, SDC sections and DEs may be repeated and nested to any level of depth.

The structure of SDC is defined by a set of nested eXtensible Markup Language (XML) schemas. The schemas constrain the structure of SDC XML to recurring patterns and are also used to generate programming code to create the SDC Object Model (OM). The OM is used to generate SDC XML from SDC modeling tools and may also be used to control the behavior of SDC-based DEFs. Details about the SDC Schema set may be found in the SDC Technical Reference Guide.^9,19^

**FIG 1.**
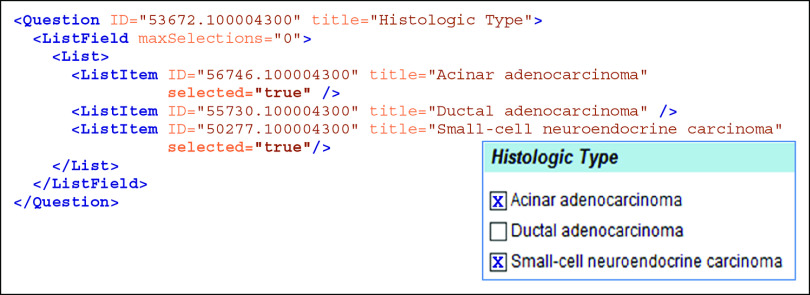
SDC data element. The figure shows an example of an SDC XML data element corresponding to a multiselect question, with the matching part of an eXtensible Stylesheet Language with Transformation-generated HTML DEF shown in the inset (lower right). Each of the 3 answer choices in the DEF inset matches to a ListItem in SDC XML. ListItem elements are nested under the Question element with wrapper elements in the sequence Question → ListField → List→ {ListItems}, where {ListItems} represents the list of ListItem elements. As shown in the Histologic Type DEF (inset), a user has selected the first and third answer choices. In the SDC XML, a selected answer choice is expressed with the selected = “**true**” expression on the corresponding ListItem elements. The expression maxSelections = “0” on the ListField element indicates that the Question is multiselect. Note that each Question and ListItem has a unique ID attribute. The .100004300 part of each ID is the namespace designation for the College of American Pathologists. DEF, data entry form; ID, identifier; SDC, Structured Data Capture; XML, eXtensible Markup language; XSLT, eXtensible Stylesheet Language with Transformations. (From NAACCR Volume V,^27^ with slight modification. Used with permission from NAACCR.)

SDC XML documents (Fig 1) that are used to generate DEFs are called Form Design Files (FDFs). An FDF may be converted to a DEF using a variety of techniques. One popular technique is to use a program (often written in eXtensible Stylesheet Language with Transformations [XSLT]) to convert the FDF into a functional web page, with JavaScript controllers to implement SDC rules and data submission functionality. However, most vendors who support SDC do not use webpages, but instead use proprietary techniques to transform the FDF into their preferred software implementation.

### Common Data Elements and Terminologies

SDC can also define common data elements (CDEs). CDEs are DEs that are common across multiple data sets and/or are shared across clinical domains and/or reused in many different FDFs.^20,21^ SDC’s use of CDEs provides an important layer of data interoperability and reuse by predefining sharable DEs that are needed for the FDF clinical content.^22^

Ideally, CDEs should be paired with appropriate standard terminologies to optimize interoperability and encourage CDE reuse.^23^ Terminology standards are critical to provide the semantic meaning and context of CDE components when CDEs are separated from their SDC source, when used by analysts who may not have access to the SDC or CDE definition, or when combining with data sets from non-SDC and/or non-CDE sources. Similarly, SDC DEs also benefit from being mapped to standard terminologies.

The SDC content management workflow is improved by using ancillary SDC mapping files for CDEs and terminologies, rather than placing CDE and terminology metadata directly in FDFs. External FDF maps promote centralized mechanisms for terminology management, validation, distribution, and searching for new and updated code sets, and they also enable the transmission of smaller SDC messages. However, some use cases may require the transmission of terminology codes within the FDF, and SDC supports this model as well.

### SDC + Systematized Nomenclature of Medicine Clinical Terms

**FIG 2.**
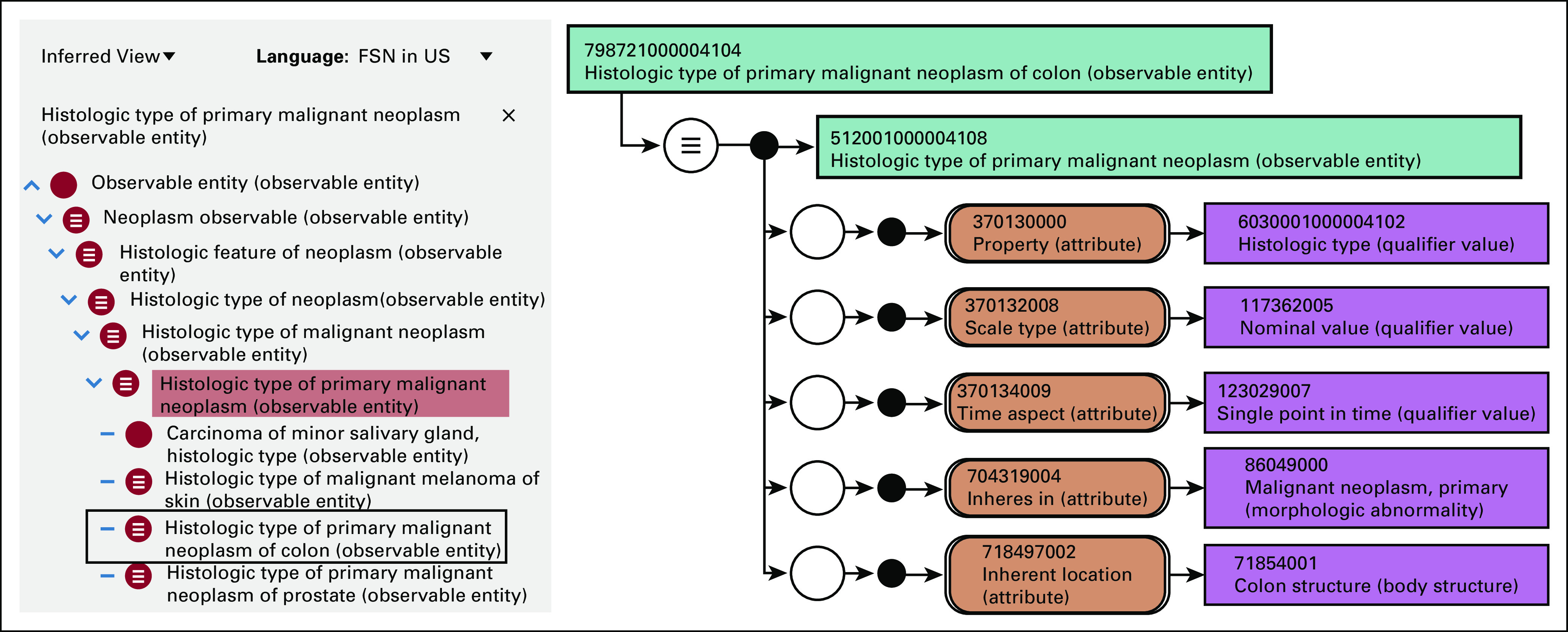
SNOMED CT modeling designed for use with SDC and common data element–based data analysis. The figure shows the SNOMED CT concept for *Histologic type of primary malignant neoplasm of colon*. The right portion of the figure shows the concept’s stated definition (ie, the definition provided by the concept author), which indicates that it is an observation of the histology type of a primary malignant neoplasm located in the colon made at a single point in time. The left portion of the figure, inside the grey rectangle, represents the classified concept definition (ie, the augmented definition produced from the SNOMED CT description logic classifier), which asserts that the observation is a subtype of observation of histology of primary malignant neoplasm and several other higher-level concepts. Furthermore, the concept is grouped, or aggregated, with all other types of observations of histologic types of primary malignancies regardless of organ, such as prostate and melanoma. This classified definition supports data queries such as “find any instance of adenocarcinoma in any organ” or “find all histologic types associated with primary colon tumors.” SDC, Structured Data Capture; SNOMED CT, Systematized Nomenclature of Medicine Clinical Terms.

Systematized Nomenclature of Medicine Clinical Terms (SNOMED CT) is the most comprehensive controlled medical terminology and is broadly adopted internationally. SNOMED CT is polyhierarchical, allowing multiple parent nodes per clinical concept. It is composed of 19 domain hierarchies, such body structure, observable entity, and clinical finding. Each concept may be defined (rendered computable) by specifying supertype(s) and additional defining attributes from the various domain hierarchies. Defined concepts are subjected to computer classification, which moves each concept under its logical parent concepts and creates additional logical concept relationships. The result is a robust searchable ontology that allows for granular, specific concept definitions, concept aggregations, and concept grouping by defining characteristics.^24^ In 2014, investigators at the University of Nebraska Medical Center began development of SNOMED CT concepts specific to the eCC SDC content to address terminology deficiencies noted by the Centers for Disease Control and Prevention (CDC) for cancer reporting.^25,26^ An example of the new SNOMED CT modeling for the eCC SDC templates is provided in Figure 2.

SDC IDs for answer choices change whenever the answer choice semantics change. For SDC questions, the SDC ID changes whenever any change is made to the semantics of the question or any of its child answer choices. This provides clear documentation whenever the DE’s composite semantics changes. However, this level of semantic version control in SDC can be undesirable when stable IDs are desired for querying across DE versions, where SDC IDs are used as query targets. SNOMED CT, when mapped to each SDC question and answer, solves this problem by providing stable semantic IDs for each SDC ID. Additionally, the SNOMED CT ontology provides new opportunities for analytics such as increasing or decreasing the granularity of queries through drilldowns and rollups, which would be impossible with SDC IDs alone. Finally, SNOMED CT provides a robust analytics capability that can survive minor DE version changes that alter SDC IDs.

### SDC Data Transmission

When an SDC DEF is filled out, the user’s responses may be stored inside the FDF XML, which is now called an FDF with Responses (FDF-R). The FDF-R may undergo cycles of edit-save-edit revisions before being transmitted (using standard IHE transactions) to one or more end points, such as EHRs and public health agencies. Alternatively, responses may be extracted from the DEF or FDF-R and transmitted in any suitable format, such as North American Association of Central Cancer Registries (NAACCR) Volume V,^27^ which uses Health Level Seven International (HL7) v.2.5.1, or IHE SDC on FHIR^28^ (discussed below). While recreating the transmitted DEF at the end point node is a trivial task if the FDF-R is transmitted intact, the question and answer responses can be extracted and reconstructed into an SDC DEF after using any of the above transmission techniques.

### SDC on FHIR

The IHE SDC working group is developing a transmission specification for IHE SDC using FHIR as the wrapper or transport mechanism for SDC forms.^28^ This approach wraps or converts SDC forms to a variety of FHIR resources. A FHIR resource is a reusable data structure that represents a small domain of healthcare information. Examples include patient, practitioner, claim, and location. IHE SDC on FHIR uses the resources named DocumentReference and Observation.^29,30^ This approach provides seamless interoperability between FHIR and IHE SDC. SDC forms and data are transported in a FHIR DocumentReference wrapper, and the FDF-R question and answer content is parsed into individual FHIR Observation objects.^31^ The SDC Observations can be processed and queried like any other FHIR data, expanding the downstream usability of the SDC data.

SDC DEs and FHIR Observations both support repurposing of SDC data for reuse in other types of data sets, eg, biospecimen annotations, clinical trial forms, reports, and rules engines. The SDC IDs and mapped terminology codes allow downstream systems to reconstruct the semantic, contextual, and structural aspects of the DEs, and if required, to trace back to the SDC form where the data originated.

## SDC ADOPTION IN CANCER PATHOLOGY

The standardization of SDC features across implementers allows accreditation organizations to support their requirements through interoperable, metadata-driven content and behavior. The extent of SDC adoption can be gauged by the number of licensed users of CAP eCCs. Currently, 45% of hospitals with > 400 beds in the United States are licensed to use the eCC.^10^ In addition, 92% of Ontario, Canada pathologists were using the eCCs as of 2012, and according to the Cancer Care Ontario website, 100% of Ontario pathologists are currently using the eCCs, now released only in SDC format.^3,32,33^

## SDC RESEARCH AND DEVELOPMENT

### SDC-Based Breast Cancer Staging Calculator

One important example of new feature testing involves the implementation of an SDC-based Breast Cancer Staging Calculator (BCSC) that uses the American Joint Committee on Cancer (AJCC) staging application programming interface. New features piloted in the BCSC reference implementation included a more advanced use of skip logic (turning DEF parts of/off depending on the user type [pathologist or oncologist]) the use of surrogate codes in a format required by the staging web service, the aggregation of parameter values from selected answers and user-entered values, the sending of those parameter values to a staging web service (created by AJCC and CDC), return of the values to designated parts of the SDC DEF, generation of a full synoptic report on the basis of the user responses and values returned from the staging web service, and also transmission of that report to a CDC server using the IHE SDC SubmitForm transaction.^9^ These features are specified declaratively inside the FDF XML, without any procedural code. In the BCSC reference implementation, small JavaScript services were used to read the FDF XML metadata and implement the above behavior when DEF buttons were pressed and when the form results were submitted to the CDC server. This pilot served as a demonstration of a multipart SDC form that is used by three different physicians in sequence to produce an integrated staging report for automating both clinical and pathological AJCC staging. Introducing these features for vendor implementation would likely require 1-2 years of additional work after the project plan is approved by the various stakeholders.

### Computable Care Guidelines

The Computable Care Guideline (CCG) technical framework reinterprets written guidelines as interoperable computer operations.^34,35^ The technical framework is based on the FHIR Clinical Practice Guidelines Implementation Guide by the HL7 Clinical Reasoning Work Group.^36^ In a CCG, data are collected by SDC form components, which are used to trigger FHIR-based rule blocks called Cards, on the basis of FHIR ActivityDefinition.^37^ Cards are connected to each other using SDC-derived responses and mapped terminology codes transmitted as FHIR transactions.

For example, a cancer diagnosis or staging guideline can be converted to a set of cascading SDC forms and cards that communicate with an EHR system. As clinical results from the SDC form are saved into the patient’s health record, card instructions will present EHR notifications to appropriate members of the care team with the next steps for their patient.^38,39^ The DEs inside the SDC forms were mapped to terminology codes that enabled coordination between the DEFs, cards, and the EHR in the demonstration.

### Computer-Assisted Reporting and Decision Support (CAR/DS)

The CAR/DS framework (no relationship to CCG Cards) is a machine-readable XML-based definition format for representing radiology reporting clinical guidelines created by the American College of Radiologists.^40^ Like SDC, CAR/DS is also designed to be an open framework for the creation of additional guidelines. The CAR/DS and SDC groups are developing a pilot to correlate radiology (CAR/DS) and pathology (SDC) cancer diagnoses through the use of FHIR transactions.^41^ Because CAR/DS and SDC both can capture data at the point of care and then convert them to FHIR Observations, they can be made interoperable through FHIR technology. By mapping common DEs across CAR/DS and SDC templates, radiologists will be able to automate the creation of concordance reports that compare the radiologic diagnosis with the pathologic diagnosis. Concordance reports help radiologists determine the accuracy of their radiographic assessments. A pilot project between American College of Radiologists and CAP is applying this approach to the Thyroid Imaging Reporting & Data System (TI-RADS) and the Thyroid eCC SDC template.

### Registry Data Dictionaries and Form Templates

NAACCR maintains an extensive composite data dictionary, known as NAACCR Volume II, for cancer registries.^42,43^ This data dictionary is complex, containing generic DEs applicable to all tumor types and Site-Specific Data Items applicable only to certain tumors.^44^ Frequent NAACCR volume II changes are a challenge for the various kinds of software that must be updated and the systems that must analyze changing data sets. Furthermore, NAACCR volume II must maintain compatibility with other cancer data standards such as the eCCs, AJCC staging, and the International Classification of Diseases for Oncology, third edition (ICD-O-3) cancer classification system. The CAP and the CDC are exploring ways to represent the NAACCR Volume II DEs in SDC format, on a tumor-specific bases, with one FDF per tumor type. The tumor pathology would be drawn largely from existing eCC CDEs, which are already harmonized with AJCC staging, ICD-O-3 and other clinical standards. In this pilot model, each FDF would serve as both a data dictionary and as a DEF template for registry software vendors, potentially alleviating some of the NAACCR versioning challenges.

### Minimal Common Oncology Data Element

The minimal Common Oncology Data Elements (mCODE) project curates a standardized set of oncology data represented as FHIR resources. mCODE resources are designed to promote data transfer between EHRs and other systems such as registries and clinical trials software. mCODE began as a collaboration between ASCO and the MITRE Corporation.^45^ mCODE data may be collected using a variety of tools, including SDC-based DEFs. Current work is exploring ways to transmit SDC-collected data as mCODE resources. Future work may include mapping from specific SDC DEs to mCODE FHIR resources. Additionally, mCODE DEs may be combined with SDC data using FHIR bundles to create a more comprehensive report.

### Other Ongoing SDC Work

**FIG 3.**
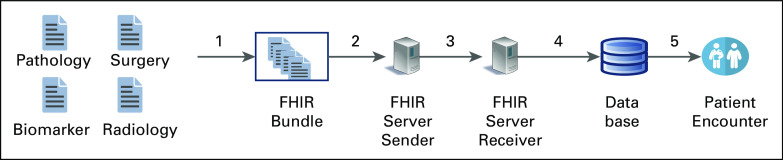
SDC on FHIR. The figure shows submission of several SDC forms with SDC on FHIR, using FHIR DocumentReference and Observation in a Bundle. Arrow 1 shows a group of SDC forms being processed for inclusion in an FHIR bundle. Arrow 2 shows the use of SDC on FHIR to submit the FHIR bundle from a sending server to a receiving server. Arrow 3 shows submission of the SDC on FHIR bundle to a receiver. Arrow 4 shows submission to a database end point. Arrow 5 shows the extraction, transformation, and transfer of that data to permit viewing by end users. SDC, Structured Data Capture.

Additional ongoing SDC work involves testing of SDC schema enhancements, improving documentation, and piloting new approaches to data bundling, transmission, and downstream use. Examples include pilots using SDC + FHIR bundles containing arbitrarily complex SDC and FHIR content,^43^ tools to extract and display the transmitted data, bulk data transmissions to facilitate large data sets in public health use cases (Fig 3),^46,47^ sharing patient data with multiple members of a distributed care team in diverse locations, and automated trigger-based clinical decision support.

In conclusion, IHE SDC is a technology designed to meet both data capture and interoperability needs. Complex, frequently changing, oncology data sets were a major design focus. SDC synergizes with technologies such as FHIR and SNOMED CT to greatly increase the capabilities of standardized structured reporting. Some capabilities include improving guideline adherence, clinical decision support, public health surveillance, and data aggregation for research and clinical trials.

Although most vendors who use SDC opt for desktop application DEF technology, SDC-enabled DEF software is also straightforward to implement using standard web technologies. An open-source, web-based SDC reference implementation is available on the IHE-SDC-WG GitHub.^48,49^ Because of its flexible and interoperable features, SDC could play an important role in data capture for healthcare by creating centrally standardized DEFs for diseases like cancer and could be leveraged for emerging diseases such as COVID-19.
